# Optimized Protocol for Characterization of Mouse Gut Innate Lymphoid Cells

**DOI:** 10.3389/fimmu.2020.563414

**Published:** 2020-11-30

**Authors:** Ana Valle-Noguera, María José Gómez-Sánchez, Mathilde J. H. Girard-Madoux, Aranzazu Cruz-Adalia

**Affiliations:** ^1^Department of Immunology, School of Medicine, Complutense University of Madrid, 12 de Octubre Health Research Institute (imas12), Madrid, Spain; ^2^Department of Molecular & Cellular Biology, Centro Nacional de Biotecnología, Consejo Superior de Investigaciones Científicas (CNB-CSIC), Madrid, Spain; ^3^Centre d’Immunologie de Marseille-Luminy, Université d’Aix-Marseille UM2, Inserm, U1104, CNRS UMR7280, Marseille, France

**Keywords:** innate lymphoid cells, flow cytometry, small intestine, colon, lamina propria cells, innate lymphoid cell type 3, gut immunology, ILC3 cytokines

## Abstract

Since their discovery, innate lymphoid cells (ILCs) have gradually been gaining greater relevance in the field of immunology due to their multiple functions in the innate immune response. They can mainly be found in mucosal and barrier organs like skin, gut, and lungs, and have been classified into five main types (NKs, ILC1s, ILC2s, ILC3s, and Lti cells) according to their function and development. They all play major roles in functions such as tissue homeostasis, early pathogen defense, regulation of inflammation, or tissue remodeling. ILCs are mostly tissue-resident cells tightly bound to the tissue structure, a fact that requires long and complex protocols that do not always provide sufficient yield for analysis. This suggests the need for optimized approaches aimed at ensuring that enriched and viable ILC samples are obtained, in order to furnish quality results. Herein a detailed protocol is established for obtaining a single-cell suspension highly enriched in lymphoid cells from mouse gut in order to identify the different subsets of ILCs by means of flow cytometry. The cell marker panel and flow cytometry gating strategies for identification and quantification of all the different ILC populations are provided for simultaneous analysis. Moreover, the protocol described includes a procedure for studying the different cytokines produced by ILC3s involved in maintaining the integrity of the gut barrier and defending against extracellular pathogens. As a result, herein an efficient method is presented for studying mouse ILCs within the lamina propria of the small intestine and colon; this can constitute a useful tool for future investigations in the field.

## Introduction

The Innate Lymphoid Cells (ILCs) are a recently discovered subset of immune cells that play a unique role at the interface between the immune system and the mucosal environment. Based on mouse and human model studies, ILCs evolve from common innate lymphoid progenitors (CILPs) into natural killer (NK) cell precursors that differentiate into NK cells or into common helper innate lymphoid progenitors (CHILPs); the latter in turn give rise to lymphoid tissue inducer cell (Lti) precursors (LtiPs) and ILC precursors (ILCPs). LtiPs differentiate toward Lti cells and ILCPs give rise to ILC1s, ILC2s and ILC3s (1–5). Each stage of differentiation is driven by the activation of different transcription factors which in the last step are essentially eomesodermin (Eomes) for NK, T-bet for ILC1, GATA3 for ILC2, and ROR*γ*t for ILC3 and Lti cells (6).

**Figure 1:**
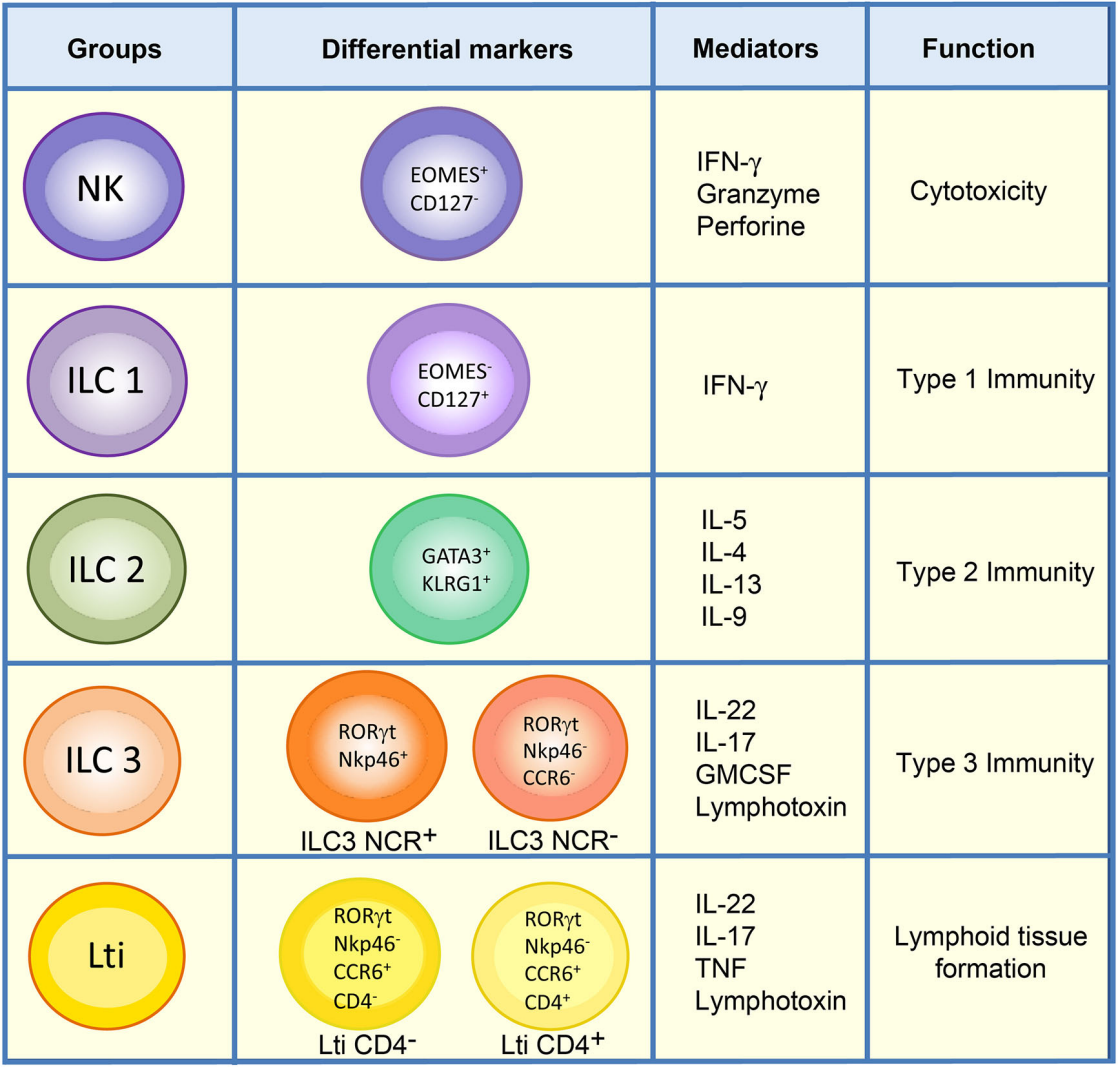
Classification of ILCs. ILCs are classified into five groups: NKs, ILC1s, ILC2s, ILC3s, and Lti cells. ILC1 and NK populations in the lamina propria of mouse intestine can be differentiated by the markers Eomes and CD127. Unlike ILC1s, NKs are mainly cytotoxic, and both types of cells direct type 1 immunity though the secretion of cytokines such as IFN-*γ*. ILC2s express GATA3 and KLRG1 and are involved in type 2 immunity. The ILC3 group comprises ILC3 NCR^+^, which expresses Nkp46 in mouse, and ILC3 NCR^-^. They mediate type 3 immunity along with Lti cells, which can be distinguished from ILC3s by the expression of CCR6. Lti cells are highly relevant in the formation of lymphoid structures, and in adult mouse gut either CD4^+^ Lti or CD4^-^ Lti cells can be found.

The main characteristic of ILCs is that they are antigen receptor-negative lymphocytes (B cell receptor and T cell receptor) that efficiently produce cytokines (Interferon gamma (IFN-*γ*), interleukin (IL)-5, IL-13, IL-17A, IL-22) upon activation (7). Although the initially proposed classification of ILCs divided them into three different subtypes of cells according to their resemblance to T cells (8), a new revised nomenclature has been approved by the International Union of Immunological Societies, in which ILCs can be classified into five subsets: NKs, ILC1s, ILC2s, ILC3s, and Lti cells, according to their development and function (6) (**Figure 1**).

NKs are cytotoxic cells whose function involves killing virus-infected and tumor cells (9, 10). Accordingly, NKs can be considered as the innate counterpart of CD8^+^ T cells. NK cells share many features with ILC1s, including expression of the transcription factor T-bet, required for their function, and the production of IFN-*γ* as their principal cytokine output. However, unlike ILC1s, NK cells express Eomes and generally lack expression of CD127 (IL-7R α) (6) (**Figure 1**). ILC1s are broadly non-cytotoxic and have similar characteristics to T helper 1 (Th1) cells; they secrete IFN-*γ* and the tumor necrosis factor (TNF), both of which are vital for combating intracellular pathogens, such as *Salmonella typhimurium, Listeria monocytogenes, Toxoplasma gondii* and viruses (2, 11–13). ILC2s mimic the function of Th2 cells and respond to large extracellular parasites, such as *Nippostrongylus brasiliensis*, and allergens by secreting IL-13, IL-4, and IL-5 (11, 14–17). ILC2s express larger amounts of the transcription factor GATA3 than the other ILC subsets, and the absence of this transcription factor abrogates the development and function of these cells. They share with other ILC populations the expression of a number of activating and inhibitory receptors such as KLRG1 (**Figure 1**).

ILC3s are similar to Th17, combating extracellular microorganisms, such as bacteria like *Citrobacter rodentium*, and fungi (8, 18–20). Furthermore, ILC3s help to maintain the gut microbiota by secreting IL-17 and IL-22, which stimulate intestinal endothelial cells (IEC) to produce antimicrobial peptides and mucins (RegIIIβ, RegIIIγ, S100A8, and S100A9) (18, 21–28). ILC3s constitute the largest population of ILCs in the gut in steady state and are essential for the maintenance of gut homeostasis (28). They express the nuclear receptor ROR*γ*t necessary for their generation and function, and comprise two subsets of cells defined by their cell surface expression of cytotoxicity receptors (NCR), NKp46 (in mice), or NKp44 (in humans) (6, 22, 29) (**Figure 1**). Lymphoid tissue inducer cells are essential for the organogenesis of lymph nodes during fetal development and for host innate immunity (18, 30). They also produce substantial amounts of IL-22, as well as IL-17 if stimulated with IL-23 (31), to protect against extracellular bacteria and fungi. In mice, Lti cells can be distinguished from other ILC3s by their expression of the chemokine receptor CCR6 (32). Some Lti cells can also express CD4, and these are considered to constitute the most mature Lti cells (**Figure 1**). Nonetheless, a gene expression analysis of both populations in the lamina propria of mouse small intestine revealed no substantial differences between them (33).

Most of the ILCs are found mainly in the mucosa and mucosal-associated lymphoid tissues, where they rapidly initiate immune responses to pathogens without the need for specific sensitization (14). These tissue-resident cells have been studied mainly in the lungs and gut in multiple immune pathways and pathologies such as cancer, allergies, asthma, arthritis, obesity, psoriasis, and Inflammatory Bowel Disease (IBD), and also in diverse functions like tissue remodeling, inflammation regulation and crosstalk with the nervous system (6, 34–36).

The importance of the study in gut ILCs lies in the strong connection between gut homeostasis and overall body health. Gut malfunction and dysbiosis have been proven to be linked to a variety of pathologies like ulcerous colitis, Crohn’s disease, or colon carcinoma (37, 38). ILCs, especially ILC3s have recently been discovered to play an important role in controlling and fighting these pathologies, mainly due to their interaction with microbiota and IL-22 and IL-17 cytokine release (6). Consequently, there is a vital need to investigate their functions, interactions, and the molecular mechanisms at play in homeostasis and during the development of certain diseases.

As gut ILCs are deeply embedded in the extracellular matrix of the tissue, isolation and analysis thereof require a complex multistep protocol. This research expounds a step-by-step procedure for enriching and analyzing the different populations of ILCs from the lamina propria of both the murine colon and small intestine. Furthermore, the paper provides a flow cytometry strategic analysis of ILCs and the quantification of gut ILC3 cytokine production, which could be useful for further study and characterization of the function of these subsets of cells.

## Methods

### Animals

C57BL/6 mice were bred and housed in a climate-controlled environment at the National Biotechnology Center in Madrid (CNB) following the sanitary recommendations of the Federation of European Laboratory Animal Science Associations (FELASA). Animals had *ad libitum* access to a standard mouse chow diet and water. All experimental procedures were approved by the Animal Care and Ethics Committee of the CNB and by the regional authorities (project n° PROEX 146/18).

### Colon and Small Intestine Processing

#### Isolation of Colonic Lamina Propria Cells

Mice were sacrificed by means of cervical dislocation or administration of CO_2_ and transcardially perfused with 10 ml of Phosphate Buffered Saline 1X (PBS). The colon was harvested and cleaned of fat residue and feces. To obtain lamina propria cells (LPCs), the colon was cut longitudinally and fragmented into pieces; it was then placed in Colon Predigestion Medium and incubated at 37°C to remove epithelial cells and was vigorously shaken for 30 min. The supernatant was discarded and the samples were washed 4 times with PBS 1X to remove ethylenediaminetetraacetic acid (EDTA); they were then incubated in Colon Digestion Medium with collagenase (Sigma) to disaggregate the tissue by means of vigorous shaking for 45 min. The LPCs were then enriched with the use of a percoll gradient.

Note: Conserve the colon in ice-cold PBS 1X after harvesting and prior to processing the tissue.

#### Isolation of LPCs From the Small Intestine

The mice were sacrificed by cervical dislocation or CO_2_ administration and transcardially perfused with 10 ml of PBS 1X. The small intestine (SI) was harvested and cleaned of fat residue and Peyer’s patches. To obtain LPCs, the small intestine was cut longitudinally and scraped in PBS 1X to clean the mucus and feces. The clean tissue was fragmented into pieces in SI Predigestion Medium and incubated in a shaker at 37°C for 30 min to remove epithelial cells. The supernatant was discarded and the samples were washed 4 times with PBS 1X to remove EDTA; they were subsequently incubated in SI Digestion Medium with collagenase (Sigma) for 20 min in a shaker at 37°C in order to disaggregate the tissue. The LPCs were then enriched with a percoll gradient. The different steps for the colon and the small intestine protocols are indicated in **Table 1**.

**Table 1:** Differences in the steps of the protocols for colon and small intestine.

Step	Colon protocol	Small intestine protocol
Cleaning	-Cleaning the fat residue and feces with curved forceps	-Cleaning the fat residue with curved forceps - Rolling in tissue paper - Elimination of Peyer’s patches - Scraping to clean the mucus and feces
Predigestion	EDTA treatment	EDTA and DTT treatment
Washing	Similar	Similar
Enzymatic digestion	Collagenase treatment	Collagenase, DNAse and Dispase treatment
Percoll gradient	Similar	Similar

Note: Conserve the small intestine in ice-cold PBS 1X after harvesting and prior to processing the tissue.

#### ILC3 Activation for Cytokine Analysis

This is an optional step for analyzing the cytokine secretion of the ILC3s. Lamina propria single-cell suspensions were cultured and stimulated at a density of 1M/ml in culture medium (RMPI, 10% fetal bovine serum (FBS) and 1% penicillin/streptomycin) with IL-23 (R&D) (40 ng/ml), IL-1β (StemCell) (100 ng/ml) and Brefeldin A (Cell Signaling) (5 µg/ml) for 4 h at 37°C.

### Flow Cytometry Analysis of ILCs

Following centrifugation in percoll, the cells were washed with PBS 1X and incubated with Fc Block (anti CD16/CD32; BD Bioscience) and rat serum (1/100) in PBS 1X for 10 min at 4°C. Subsequently, the LPC single-cell suspensions were stained with the following anti-mouse membrane protein antibodies (Abs) at 37°C for 45 min: anti CD45.2 PerCP-Cy 5.5 (BD Bioscience), anti CD127 PE-Cy5 (eBioscience), anti CD335 (NKp46) PE-Cy7 (eBioscience), anti CD4 AlexaFluor 700 (eBioscience), anti KLRG1 APC eFluor 780 (eBioscience), anti CD3e BV421 (BD Bioscience), anti CCR6 APC (Biolegend), anti CD19 BV421 (BD Bioscience), anti Ly-6G and Ly-6C (GR-1) BV421 (BD Bioscience), anti F4/80 BV421 (BD bioscience), anti TCR β BV421 (Biolegend), anti TCR *γ*δ BV421 (Biolegend), anti CD5 BV421 (Biolegend), and the Live/Dead Fixable Yellow dead cell stain (Thermo Fisher). They were then fixed and permeabilized with the Foxp3/Transcription Factor Staining Buffer Set (Thermo Fisher Scientific) following the manufacturer’s guidelines for 96-well plates. Finally, they were stained with the following anti-mouse transcription factor Abs at room temperature for at least 1h: ROR*γ*t PE (eBioscience), anti Eomes PE-eFluor 610 (eBioscience), and anti GATA-3 Alexa Fluor 488 (BD Bioscience). **Table 2** indicates all the antibodies required for this flow cytometry panel, along with the recommended dilution of use.

**Table 2:** Protein markers for analysis of ILC populations by flow cytometry.

Antigen	Clone	Fluorochrome	Brand	Dilution
CD45.2	104	PerCP-Cy 5.5	BD	1/100
CD127	A7R34	PE-Cy5	eBioscience	1/100
CD335 (Nkp46)	29A1.4	PE-Cy7	eBioscience	1/50
CD4	GK 1.5	Alexa Fluor 700	eBioscience	1/100
CCR6	29-2L17	APC	Biolegend	1/100
KLRG1	2F1	APC-eFluor 780	eBioscience	1/100
CD3	145-2C11	BV421	BD	1/200
CD19	1D3	BV421	BD	1/100
Ly-6G and Ly-6C (GR-1)	RB6-8C5	BV421	BD	1/100
F4/80	T45-2342	BV421	BD	1/200
TCR β	H57-597	BV421	Biolegend	1/100
TCR *γ*δ	GL3	BV421	Biolegend	1/100
CD5	53-7.3	BV421	Biolegend	1/100
*GATA-3	L50-823	Alexa Fluor 488	BD	1/50
*RORγt	AFKJS-9	PE	eBioscience	1/50
*Eomes	Dan11mag	PE-eFluor 610	eBioscience	1/100
CD16/CD32 (Fc block)			BD	1/200

*Transcription factors.

#### Flow Cytometry ILC3 Cytokine Analysis

Following activation, cells were washed with PBS 1X and incubated in Fc Block and rat serum in PBS 1X for 10 min at 4°C. For surface staining, the following anti-mouse membrane protein Abs were used at 37°C for 45 min: anti CD45.2 PerCP-Cy 5.5 (BD Bioscience), anti CD335 (NKp46) PE-Cy7 (eBioscience), anti CD4 AlexaFluor 700 (eBioscience), anti CD3 PE-Cy5 (Tonbo), anti CD19 FITC (Tonbo), anti Ly-6G and Ly-6C (GR-1) FITC (Tonbo), anti F4/80 FITC (Tonbo), and the Live/Dead Fixable Yellow dead cell stain (BD Bioscience). They were then fixed and permeabilized with the Factor Staining Buffer Set (Thermo Fisher Scientific) following the manufacturer’s guidelines for 96-well plates. Subsequently, they were stained with the following anti-mouse transcription factors and cytokine Abs at room temperature for at least 1 h: ROR*γ*t BV421 (Thermo Fisher), anti IL-22 PE (eBioscience), and anti IL-17 APC (eBioscience). **Table 3** indicates all the antibodies required for this flow cytometry panel, along with the recommended dilution of use.

**Table 3:** Protein markers for analysis of ILC3-expressing cytokines.

Antigen	Clone	Fluorochrome	Brand	Dilution
CD45.2	104	PerCP-Cy 5.5	BD	1/100
CD3	145-2C11	PE-Cy5	Tonbo	1/100
Cd335 (NKp46)	29A1.4	PE-Cy7	eBioscience	1/50
CD4	GK 1.5	AlexaFluor 700	eBioscience	1/100
CD19	1D3	FITC	Tonbo	1/100
Ly-6G and Ly-6C (GR-1)	RB6-8C5	FITC	Tonbo	1/100
F4/80	BM 8.1	FITC	Tonbo	1/200
*RORγt	Q31-378	BV421	BD	1/50
*IL-22	1H8PWSR	PE	eBioscience	1/50
*IL-17	eBiol7B7	APC	eBioscience	1/100
CD16/CD32 (Fc block)			BD	1/200

*Intracellular proteins.

## Materials and Reagents

### Materials and Equipment

- Surgical kit for dissection (scissors, forceps, curved fine point forceps, blunt/blunt surgical scissors), 4 empty tip boxes, 1 strainer (nonmetallic tea-like strainer), icebox, ice.
-Plastics: 50 and 15 ml falcons, Petri dishes, 1.5 ml Eppendorfs, 70 µm cell strainers, serological pipets, 96-well v-bottom plaque, 24-well culture plaque.
- Centrifuge (with acceleration/brake options), orbital shaker, CO_2_ incubator
- Flow cytometry FACS Canto, and FACS Aria Fusion (BD).

### Reagents

- 3L of PBS 1X
- 100% Percoll [Pure percoll (GE Healthcare) + 10% PBS 10X], 40 and 70% percoll (100% percoll diluted with PBS 1X).
- Culture medium: RPMI {GE Healthcare Hyclone, with 25mM HEPES [4-(2-hydroxyethyl)-1-piperazineethanesulfonic acid] and L-glutamine} supplemented with 10% FBS and 1% penicillin/streptomycin.
- eBioscience™ Foxp3/Transcription Factor Staining Buffer Set (Ref:00-5523-00).
- Counting beads (ref C36950; Invitrogen) and compensation beads (Ref A10497; Life Technologies).

#### Colonic LPCs

Colon Predigestion Medium: PBS 1X supplemented with 5 mM EDTA, 14 mM HEPES and 10% FBS.

Colon Digestion Medium: RPMI (GE Healthcare Hyclone, with 25 mM HEPES, L-glutamine) supplemented with 10% FBS.

Collagenase: type VIII Collagenase (Sigma). Use: 300 UI/ml. Stock preparation: dilute in Colon Digestion Medium at 2,000 UI/ml concentration. It is recommended both working on ice during collagenase dilution and preparation and keeping the aliquots at -20°C.

#### Small Intestine LPCs

SI Predigestion Medium: 1× HBSS (no Ca 2+ or Mg 2+, no Phenol Red) supplemented with 10 mM HEPES, 5 mM EDTA, 1 mM DTT (Sigma), and 5% FBS.

SI Digestion Medium: RPMI 1640 (GE Healthcare Hyclone, with 25mM HEPES, L-glutamine) supplemented with 80 UI/ml DNAse I type IV (Sigma 2,000 UI/mg), 3.94 UI/ml Dispase II (Gibco 1.9 UI/mg) and 10% FBS.

Collagenase: type VIII Collagenase (Sigma) or Collagenase IV (Sigma). Use: 300 UI/ml. It can be added as a stock solution or directly to the digestion medium just before use. Stock preparation: dilute in Colon Digestion Medium at 2,000 UI/ml concentration.

## Statistical Analysis

All statistical analyses were performed with the use of GraphPad Prism software. Statistical significance was determined by one-way ANOVA with Bonferroni multiple comparisons (P ≤ 0.05) or a two-tailed unpaired Student’s t test.

## Stepwise Procedure

### Colon Protocol

#### Tissue Processing

**Figure 2:**
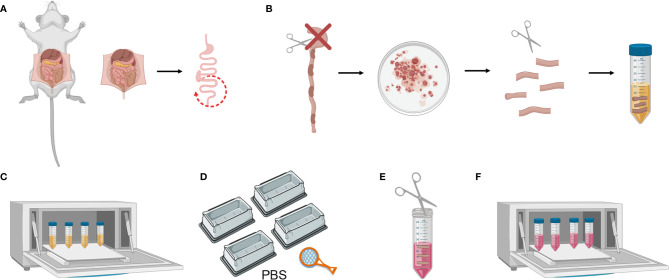
Mouse colon tissue processing. **(A)** Colon harvesting from mouse. **(B)** Washing the colon and cutting longitudinally and transversally. **(C)** Predigestion of tissue pieces at 37**°**C by shaking. **(D)** Thorough cleaning of the tissue pieces with PBS 1X. **(E)** Mincing pieces of colon with scissors **(F)** Enzymatic digestion of tissue pieces at 37**°**C by shaking.

1. First cut the femoral artery and perfuse the heart with 10 ml of PBS 1X, prior to harvesting the small intestine or colon. The lungs and liver should present a whiter appearance if the perfusion has been performed correctly. This step is critical with regard to avoiding blood in the gut, which could interfere with results.
2. Harvest the colon and keep in ice-cold PBS 1X (**Figure 2A**).
3. Clean the fat residue and feces using curved forceps. Then cut the colon longitudinally and wash it in a petri dish with PBS 1X. Cut it transversally into 1 cm-long fragments and place these in a 50 ml falcon containing 20 ml of Colon Predigestion Medium prewarmed at 37°C (**Figure 2B**).

#### Digestions

4. Shake the tissue at 200–250 rpm in an orbital shaker for 30 min at 37°C. Place the 50 ml falcons horizontally for better digestion. In this step, intestinal epithelial cells are separated from the lamina propria by means of EDTA treatment (**Figure 2C**).
5. Following incubation, invert the tubes very quickly, shaking vigorously 5–10 times to remove the remaining epithelial cells. Fill 4 tip boxes with PBS 1X and wash the tissue pieces by pouring the falcon over a strainer and shaking them in the 4 PBS-filled recipients. This is a crucial step with regard to removing the EDTA, as it interferes with the efficiency of the collagenase digestion (**Figure 2D**).
6. Collect the colon pieces in a 50 ml falcon containing 1 ml of Colon Digestion Medium and thoroughly mince them with the blunt/blunt surgical scissors (**Figure 2E**).
7. Add 16 ml of prewarmed Colon Digestion Medium and 3 ml of collagenase stock solution (Total 20 ml/colon).
8. Shake it at 200–250 rpm in an orbital shaker for 45 min at 37°C. Place the 50 ml falcons horizontally for better digestion (**Figure 2F**).

**Figure 3:**
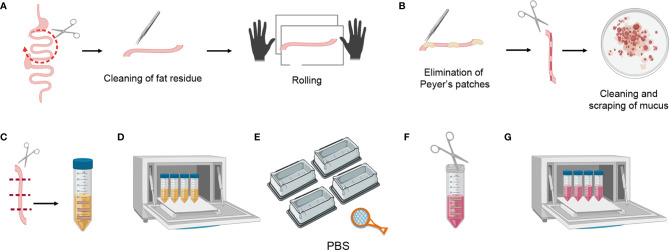
Mouse small intestine tissue processing. **(A)** Dividing the small intestine into three parts, cleaning the fat residue, and rolling. **(B)** Eliminating Peyer’s patches, cutting longitudinally, scraping the mucus, and cleaning the feces. **(C)** Transversally cutting the tissue pieces and placing them in a 50 ml falcon containing 20 ml of SI Predigestion Medium. **(D)** Predigestion of tissue pieces at 37**°**C by means of shaking. **(E)** Thorough washing of the tissue pieces with PBS 1X. **(F)** Mincing pieces of small intestine with scissors. **(G)** Enzymatic digestion of tissue pieces at 37**°**C by shaking.

### Small Intestine Protocol

#### Tissue Processing

1. Harvest the small intestine and divide it into three parts. Clean the fat residue using curved forceps, place the intestine fragment in a tissue paper and roll it to detect and clean remaining fat residue (this can be clearly seen as sticky translucent filaments) (**Figure 3A**).
2. Eliminate the Peyer’s patches and open the small intestine fragment longitudinally (this is easier to perform before the feces are cleaned). Place it in a petri dish with PBS 1X and scrape it gently to clean mucus and feces, repeat at least 3X in clean PBS 1X (**Figure 3B**).
3. Cut the small intestine transversally into 1 cm-long fragments and place these in a 50 ml falcon containing 20 ml of SI Predigestion Medium prewarmed at 37°C. Follow the same procedure for the rest of the intestine and place the fragments in the same falcon (**Figure 3C**).

#### Digestions

4. Shake the tissue at 200–250 rpm in an orbital shaker for 30 min at 37°C. Place the 50 ml falcons horizontally for better digestion (**Figure 3D**).
5. Following incubation, invert the tubes very quickly, shaking vigorously 5–10 times to remove the remaining epithelial cells. Fill 4 tip boxes with PBS 1X and wash the tissue pieces by pouring the falcon over a strainer, shaking them in the 4 PBS-filled recipients. This is a crucial step for removing the EDTA, as this interferes with the efficiency of the collagenase digestion (**Figure 3E**).
6. Collect the pieces of small intestine in a 50 ml falcon containing 1ml of SI digestion medium and mince them thoroughly with the blunt/blunt surgical scissors (**Figure 3F**).
7. Add 16 ml of prewarmed SI Digestion Medium and 3 ml of collagenase stock solution (Total 20 ml/intestine).
8. Shake at 200–250 rpm in an orbital shaker for 20 min at 37°C. Place the 50 ml falcons horizontally for better digestion (**Figure 3G**). If the intestine is not digested in 20 min, digestion time can be increased up to 40 min. Following digestion use a serological pipet to homogenize and help break up any pieces of remaining tissue.

### Colon and Small Intestine Protocol

#### Cell/Lymphocyte Purification

**Figure 4:**
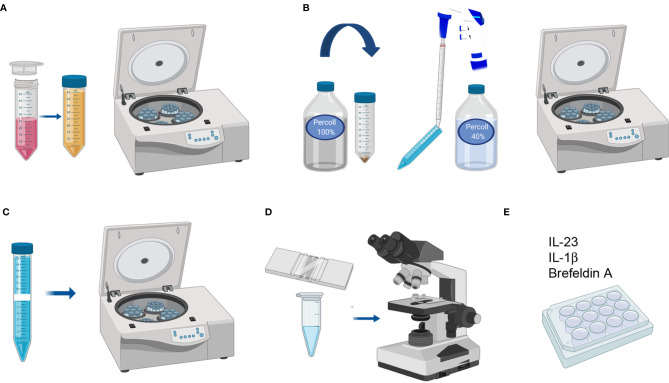
LPC isolation from colon and small intestine. **(A)** Filtering uniformly digested pieces and cleaning with Colon Predigestion Medium. **(B)** 100–40% percoll gradient centrifugation. **(C)** Collecting the middle ring of white cells and washing with PBS 1X. **(D)** Counting the cells. **(E)** Culturing the single-cell suspension with IL-23, IL-1β, and Brefeldin A for 4 h in a 24-well plate at 37°C in a CO2 incubator.

9. After incubation, filter the digested tissue through a 70 µm cell strainer (pre-wet with Colon Predigestion Medium) into a 50 ml falcon tube. Press the tissue remains against the strainer with a 10 ml syringe plunge and then wash the strainer with Colon Predigestion Medium to inactivate the collagenase (**Figure 4A**).
10. Centrifuge at 652 xg for 5 min (**Figure 4A**).
11. Discard the supernatant and place the falcon upside-down on absorbent paper to dry the pellet and resuspend the cells in 1 ml of percoll 100%. Transfer the resuspended cells into a 15 ml falcon tube and add 4 ml more percoll 100% (**Figure 4B**).
12. Add 8 ml of percoll 40% slowly from the top, letting the percoll slide down the wall of the falcon (**Figure 4B**).
13. Centrifuge at 887 xg for 20 min without acceleration or brake (**Figure 4B**).

*An alternative and more homogeneous procedure for performing the percoll gradient involves resuspending the cells in 1 ml of percoll 40% and transferring them to a 15 ml falcon tube with 3 ml more percoll 40%. Subsequently, a 70% percoll fraction (3 ml) must be underlain *via* a glass pasteur pipette followed by a 750 xg centrifugation step for 20 min without acceleration or brake (**Supplementary Figure 1**).

14. Harvest the middle ring and transfer it to another 15 ml falcon tube to wash it with PBS 1X (**Figure 4C**).
15. Centrifuge at 652 xg for 10 min and discard the supernatant.
16. Resuspend the pellet in 500 µl of Colon Digestion Medium. Cells are ready for counting and staining (**Figure 4D**).

### ILC3 Activation

1. After counting, prepare the required volume of culture medium and supplement with IL-23 (R&D) (40 ng/ml), IL-1β (StemCell) (100 ng/ml), and Brefeldin A (5 µg/ml) to culture the single-cell suspension at a density of 1 M/ml.
2. Centrifuge the single-cell suspension at 652 xg 10 min and discard the supernatant. Place the falcon upside-down on absorbent paper to dry the pellet and resuspend it in the required volume of culture medium containing cytokines. Culture in a 24-well plate for 4 h at 37°C in a CO2 incubator (**Figure 4E**).
3. Collect the cultured cells in 1.5 ml eppendorf and wash with PBS 1X.
4. Centrifuge at 652 xg for 10 min and discard the supernatant. Cells are ready to stain.

### Cytometry Staining

1. Resuspend the pellet in 50 µl/sample PBS 1X with Fc Block (1/200) and rat serum (1/100) and incubate 10 min at 4°C.
2. Transfer the cells to a 96-well v-bottom plate; remember to include an unstained cell sample and the Fluorescent Minus One (FMO), vital for optimization of flow cytometry conditions and analysis.
3. Add 50 µl/sample of PBS 1X with membrane protein Abs (consider the 50 µl from the previous step when calculating the antibody concentration) and incubate for 45 min at 37°C.
4. Wash twice with 100 µl PBS 1X and centrifuge at 652 xg for 10 min.
5. Follow the manufacturer’s guidelines for 96-well plate Foxp3/Transcription Factor Staining Buffer set from step 4 for fixation and permeabilization of samples.
6. Subsequently, centrifuge the samples at 974 xg for 10 min without brake to avoid losing cells during washes.
7. Steps 10–12 of the manufacturer’s guidelines can be substituted by adding the intracellular antibodies directly to a 50-µl sample of permeabilization buffer.
8. As the percentage of ILCs is very low, consider using compensation beads (labeled with just one of each fluorochrome used in the experiment to compensate the samples and adjust the flow cytometry settings).
9. Resuspend the cells in PBS 1X and analyze samples by flow cytometry.

## Critical Parameters and Trouble Shooting

Perform transcardial perfusions to prevent blood cells from interfering with results.
Eliminate as much fat as possible during the harvesting of the colon and small intestine for faster processing. Thoroughly remove the fat, feces, and mucus, particularly from the small intestine. N.B: this should be rolled to detect and completely remove the remaining fat residue.
For optimal digestion, immediately prior to use add the DTT, DNAse I, and Dispase II to the prewarmed solution at 37°C. Wait until complete dissolution before using. It should be noted that enzymes (especially collagenase) may provide a different yield of digestion from batch to batch; this also may vary depending on the FBS used, and enzymes and different FBS should therefore be tested prior to the experiment in order to establish the optimal ones. The digestion time of the small intestine can be increased up to 40 min if needed to complete tissue disaggregation.
After washing the percoll it is of great importance to check the tubes before discarding the supernatant; if some percoll 100% has been unintentionally added to the tube, it can form a double layer at the bottom and some cells can be accidentally discarded. If this were to occur, discard as much supernatant as possible and wash again.
Following fixation, as cells become smaller, centrifuge the samples at 974 xg for 10 min without brake to avoid losing cells during washes.
When analyzing in a flow cytometer, it is important to acquire the whole sample, because ILCs represent a very low percentage thereof.

## Results

### Characterization of the Different Subpopulations of ILCs From Colon Lamina Propria Cells

**Figure 5:**
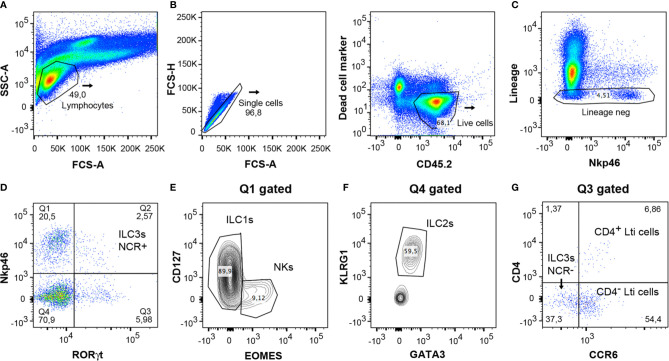
Gating strategy for analysis of innate lymphoid cells from mice colon LPCs. **(A)** Lymphocytes were gated with the forward scatter/side scatter (FSC/SSC) method. **(B)** Single and CD45.2^+^ live cells were only considered for the analysis. **(C)** Negative lineage cells were gated to analyze innate lymphocyte cells. **(D)** Expression of ROR*γ*t and Nkp46 served to distinguish the different populations of ILCs. Q1 included the NK and ILC1 populations. Q2 corresponded to the ILC3 NCR^+^ population (Nkp46^+^ cells). Q3 included ILC3 NCR^-^ and Lti cells. Q4 comprised the ILC2 population. **(E)** Staining of CD127 and Eomes was used to differentiate both populations, ILC1s and NKs, in the Q1 gated population. **(F)** Expression of KLRG1 and GATA3 served to differentiate the ILC2 cells in the Q4 gated population. **(G)** The Lti subpopulation, gated on Q3, expresses CCR6, and is divided in CD4^+^ Lti and CD4^-^ Lti cells. ILC3 NCR^-^ cells, also gated on Q3, do not express CCR6. FSC, forward scatter; SSC, side scatter.

The cells were isolated from the colon according to the protocol and were then characterized by means of multiparametric analysis with flow cytometry. For analysis of lymphocytes, they were gated with the use of the forward scatter (FCS-A) and side scatter (SSC) method (**Figure 5A**). Counting beads could be included to analyze the absolute numbers of the different cell populations. Doublet cells were excluded by plotting the height or width against the area for forward scatter or side scatter (**Figure 5B**). Subsequently, the live leukocytes were then gated with the use of the marker CD45.2 and the Live/Dead Fixable Yellow cell stain (**Figure 5B**). Cells were stained with surface markers expressed in macrophages, neutrophils, B and T cells (TCR β, TCR *γ*δ, CD5, CD3e, CD19, GR-1, and F4/80) with the same fluorochrome (indicated as lineage marker) in order to exclude them from the analysis of the ILCs (**Figure 5C**). The expression of RORγt and Nkp46 was then analyzed; this enables the different populations of ILCs to be characterized (**Figure 5D**). Cells expressing RORγt represented all ILC3 populations and Lti cells, and the expression of Nkp46 can be used to distinguish between the ILC3 NCR^+^ (Nkp46^+^) and NCR^-^ (Nkp46^-^) subsets. ILC1s and NKs were identified to be gating on Nkp46^+^ RORγt^-^ population (Q1 in **Figure 5D**) by Eomes and CD127 cell markers; Eomes^+^ CD127^-^ subset constituting the NKs and Eomes^-^ CD127^+^ population, the ILC1s (**Figure 5E**). Gating on subset RORγt- Nkp46- (Q4 in **Figure 5D**), ILC2s were distinguished by their expression of GATA3 and KLRG1 markers (**Figure 5F**). Finally, the populations of Lti and ILC3 NCR^-^ cells were analyzed through expression of the CCR6 marker (**Figure 5G**) on ROR*γ*t^+^ Nkp46^-^ gated cells (Q3 in **Figure 5D**). CD4^+^ Lti and CD4^-^ Lti cells express CCR6, whereas ILC3 NCR^-^ cells are negative for CCR6.

### Small Intestine ILC3 Yield According to Different Protocols

**Figure 6:**
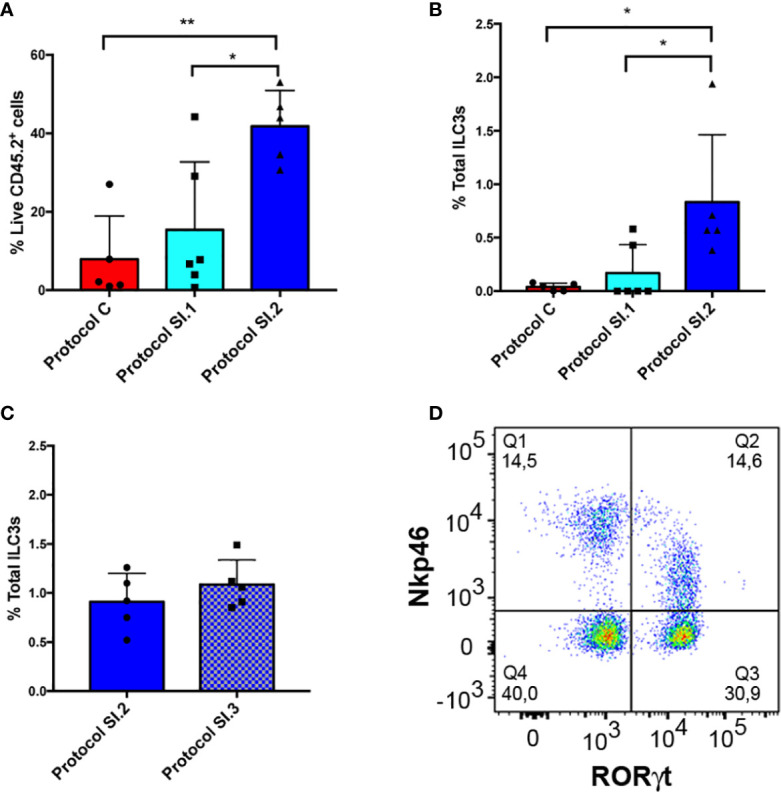
Small intestine ILC3 yield with the different protocols. **(A)** CD45^+^ live LPC yield compared among 3 different protocols. Protocol C corresponds to the LPC isolation used for the colon. Protocol SI.1 corresponds to the LPC isolation protocol used for the small intestine without considering the critical step of scraping and rolling of the small intestine. Protocol SI.2 corresponds to the protocol for the small intestine including the critical step involving thorough cleaning of the fat residue. **(B)** Comparison between the different ILC3 yields of the 3 protocols described in A. **(C)** ILC3 yield comparing protocol SI.2 with a conventional protocol based on type IV collagenase and 40–70% percoll gradient (protocol SI.3). **(D)** Representative dot plot of ROR*γ*t and Nkp46 staining of LPCs from small intestine gated on negative lineage, live CD45.2^+^ cells. Data correspond to the arithmetic mean and SD (1-way ANOVA and Bonferroni multiple comparisons test). **P < 0,005; *P < 0,05. LPCs, lamina propria cells.

Additionally, a comparison was made between the yield of live LPC CD45.2^+^ and ILC3 cells from small intestine with the use of the protocol described for colon (Protocol C, **Figure 6A**), and that established for small intestine without (Protocol SI.1, **Figure 6A**) or including (Protocol SI.2, **Figure 6A**) the critical step involving meticulous cleaning of the fat residue. Our main goal entailed improving the yield of extraction of ILCs from small intestine in order to address the very low efficiency of live CD45.2^+^ cell isolation obtained with the use of Protocol C (**Figure 6A**). Consequently, DNAase and Dispase were included in SI Digestion Medium, employed in other published protocols (39, 40), to prevent cell clumping. Unexpectedly, protocol SI.1 slightly improved the yield of LPC CD45.2^+^ and ILC3 cells (**Figure 6A, B**) but not enough to be statistically significant. However, on including the step of rolling and scraping the small intestine in order to thoroughly clean fat residue and mucus (Protocol SI.2), the percentage of isolated live LPC CD45.2^+^ and ILC3 cells exhibited a significant increase in comparison to the other protocols (**Figures 6A, B**). Furthermore, the efficiency of ILC3 isolation was analyzed vs. a conventional protocol (SI.3 protocol in **Figure 6C**), with the use of a type IV collagenase rather than type VIII in the digestion step and a 40–70% percoll gradient step (**Supplementary Figure 1**). Although the yield of ILC3 extraction was similar with both protocols (**Figure 6C**), it was much more homogenous, comfortable, and faster to perform the percoll gradient because interpersonal error was avoided by preparing the percoll with a pasteur pipette. Therefore, the different populations of ILCs could easily be characterized (**Figure 6D**). In conclusion, removing all the fat residue and mucus from the small intestine clearly constitutes a critical step for establishing the yield of live leukocytes, and consequently, for isolation of ILCs.

### Analysis of Cytokine-Producing ILC3s

**Figure 7:**
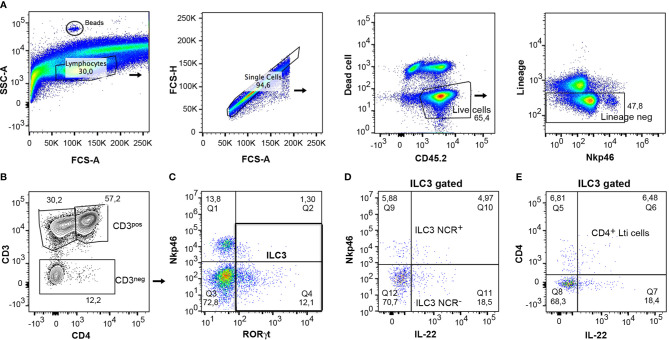
Gating strategy for analysis of mouse cytokine expressing ILC3s from colon. **(A)** CD45^+^ live cells and negative lineage were gated to analyze the different lymphocyte populations. Counting beads were included to analyze the absolute numbers of the different cell populations. **(B)** The expression of CD3 and CD4 served to differentiate the CD3^+^CD4^+^ lymphocytes, the CD3^+^ CD4^-^ lymphocytes (CD8 and *γ*δ cells), and the CD3^-^ cells, which include the innate lymphoid cells. **(C)** The CD3^-^ cells were gated and the expression of ROR*γ*t and Nkp46 was analyzed to discern between the ILC3s populations. **(D)** The expression of IL-22 was analyzed in both populations, ILC3 NCR^+^ and ILC3 NCR^-^. **(E)** IL-22-producing cells were quantified in CD4^+^ Lti cells.

Of particular interest was the study of the function of gut ILC3s due to the role they play in several diseases, such as inflammatory bowel disorders. The cells were isolated from the colon according to the protocol and were then characterized by flow cytometry. For analysis of lymphocytes, CD45.2^+^ cells were gated on live population and only lineage negative cells were included in the analysis (**Figure 7A**). The expression of CD3 and CD4 markers differentiated clearly between CD3^+^ lymphocytes and CD3^-^ cells, in which the ILCs were included (**Figure 7B**). It was then possible to analyze ILC3 and Lti cells, gating on CD3^-^ cells, as they express RORγt (**Figure 7C**). IL-22 expressing ILC3 cells were characterized in both ILC3 subpopulations, ILC3 NCR^+^ and ILC3 NCR^-^ (**Figure 7D**). A study was also conducted of IL-22 expressing CD4^+^ Lti cells gating on RORγt^+^ ILC3 cells (**Figure 7E**). This same gating strategy could be applied to small intestine samples.

To analyze the absolute number, 5 µl of CountBright absolute counting beads (ref C36950 Invitrogen) was added to each sample. They could be identified in the flow cytometer due to their high SSC-A (**Figure 7A**). To calculate the absolute number of cells/µl, follow the user’s guide and product information.

## Discussion

The present paper provides an optimized protocol for successful characterization of innate lymphoid cells from the lamina propria of mouse colon and small intestine. Moreover, a flow cytometry analysis is described for studying ILC3 expressing cytokines. Despite the fact that some published studies employ similar protocols to isolate and study lamina propria cells from gut tissue (39, 40), they are not specific or suitable for enrichment or characterization of all five populations of ILCs with the use of flow cytometry. A consistent method is developed for obtaining a high yield, which is of particular importance due to the low percentage of ILCs represented in the tissue. Isolation of LPCs consists of 5 essential steps: cleaning the fat residue and feces, EDTA-treated incubation, washing, enzymatic digestion, and percoll gradient centrifugation. The differences described between the colon and the small intestine protocols result from the fact that in the latter it is more difficult to remove the fat and that the small intestine contains a greater amount of mucus. The small intestine protocol requires the addition of other enzymes such as DNAase and Dispase in the digestion medium in order to prevent cell clumping. Despite including these modifications in the protocol, only a slight improvement was observed in the yield of LPCs and ILCs. The cleaning phase was then examined and it was found that the most crucial step with regard to achieving the desired yield in all the processed samples involved meticulous cleaning by rolling and scraping to remove fat and mucus. Moreover, the yield of ILC3 extraction was also compared with that resulting from a conventional protocol with the use of type IV collagenase and 40–70% percoll gradient, and although no difference in the percentage of total ILC3 was observed, the ease, convenience, speed, and greater homogeneity of this percoll preparation method makes it highly recommendable, since the interpersonal variable is avoided on preparing it in a pasteur pipette. In our experience, either type VIII or type IV collagenase can provide excellent digestion of the small intestine as reported by other published articles (41). The high and consistent yield obtained in all samples processed following the improved protocol for small intestine is useful for the study of ILCs, especially for research focusing on the plasticity of the populations or percentage of a specific ILC type. The protocol presented herein is also efficient for purification of all lamina propria lymphocytes.

Characterization of gut ILCs calls for a complex multiparametric flow cytometry analysis due to the lack of a unique differential cell marker (42–45). There is a current need to identify a single-cell molecule that could be used as a reliable marker, by means of either flow cytometry or immunohistochemistry. However, many ILC markers are also expressed in other immune cells, and negative lineage and transcription factor markers should therefore be included in the analysis (6, 45). It is recommended to include markers of T cells on another fluorochrome to avoid their inclusion in the ILC analysis, but if they must be included in the negative lineage due to a lack of flow cytometry canals, the use of several T cell markers, such as CD3, TCR β, TCR*γ*δ; and CD5 (46) is proposed. The flow cytometry panel and strategic gating proposed herein provide a reliable and straightforward method for studying the different ILC populations. A previously published study addresses a different flow cytometry panel used for analysing intestinal ILCs (47); this study uses T-bet as a marker of ILC1s, NKs and the subpopulation CCR6-RORγt+ within of ILC3 cells (32), but other essential markers such as NKp46 and CD4 are not included to discriminate perfectly among CD4^+^ Lti, CD4^-^ Lti, and ILC3 NCR^+^ cells. Moreover, in the present research an ILC3 secreting cytokine flow cytometric analysis is provided, which can be useful for future studies in the field.

ILC research is still in its early stages, although great progress has been made in aspects such as resistance to pathogens, regulation of chronic inflammation, tissue remodelling, cancer, and metabolic homeostasis. Several aspects of their functions as regulators of immunity, inflammation, and tissue homeostasis are still under study (48–51). The gut is one of the most scrutinized tissues, and few and outdated detailed protocols exist for isolating and analysing ILCs and their cytokines, especially for the small intestine. An optimized protocol may provide new opportunities for scientists to study a system as complex as the gut’s entire immune system.

## Data Availability Statement

The original contributions presented in the study are included in the article/**Supplementary Material**. Further inquiries can be directed to the corresponding author.

## Ethics Statement

The animal study was reviewed and approved by Animal Care and Ethics Committee of Centro Nacional de Biotecnologıa (CNB/CSIC) and the regional authorities (Comunidad de Madrid). Project n°PROEX 146/18. All animal procedures conformed to EU Directive 2010/63EU and Recommendation 2007/526/EC regarding the protection of animals used for experimental and other scientific purposes, enforced in Spanish law under Real Decreto 1201/2005.

## Author Contributions

AV-N performed and analyzed most of the experiments and helped with the manuscript writing. MG-S performed the experiments and helped in the design of some figures. MG-M helped with the design of the experiments and the revision of the manuscript. AC-A designed, supervised, and analyzed the experiments, and wrote the manuscript. All authors contributed to the article and approved the submitted version.

## Funding

The present research was supported by grant Nº RTI2018-093647-B-I00 to AC-A from Ministerio de Ciencia, Innovación e Universidades (MCIU), Agenda Estatal de Investigación (AEI), and Fondo Europeo de Desarrollo Regional (FEDER). AV-N is a recipient of an FPI fellowship (PRE2019-090341) from the Spanish Ministry of Science, Innovation, and Universities.

## Conflict of Interest

The authors declare that the research was conducted in the absence of any commercial or financial relationships that could be construed as a potential conflict of interest.

## Supplementary Material

Supplementary Figure 1 Percoll gradient step. After the digestion step, cells can be resuspended in 4 ml of percoll 40%, and 3 ml of percoll 70% must be underlain *via* a glass pasteur pipette followed by a centrifugation step of 750 xg for 20 min.
