# Supplementary figures and images for: Optimized Protocol for Characterization of Mouse Gut Innate Lymphoid Cells
Source: Front Immunol. 2020 Nov 30;11:563414. doi: 10.3389/fimmu.2020.563414 (PMC7735015; doi:10.3389/fimmu.2020.563414)

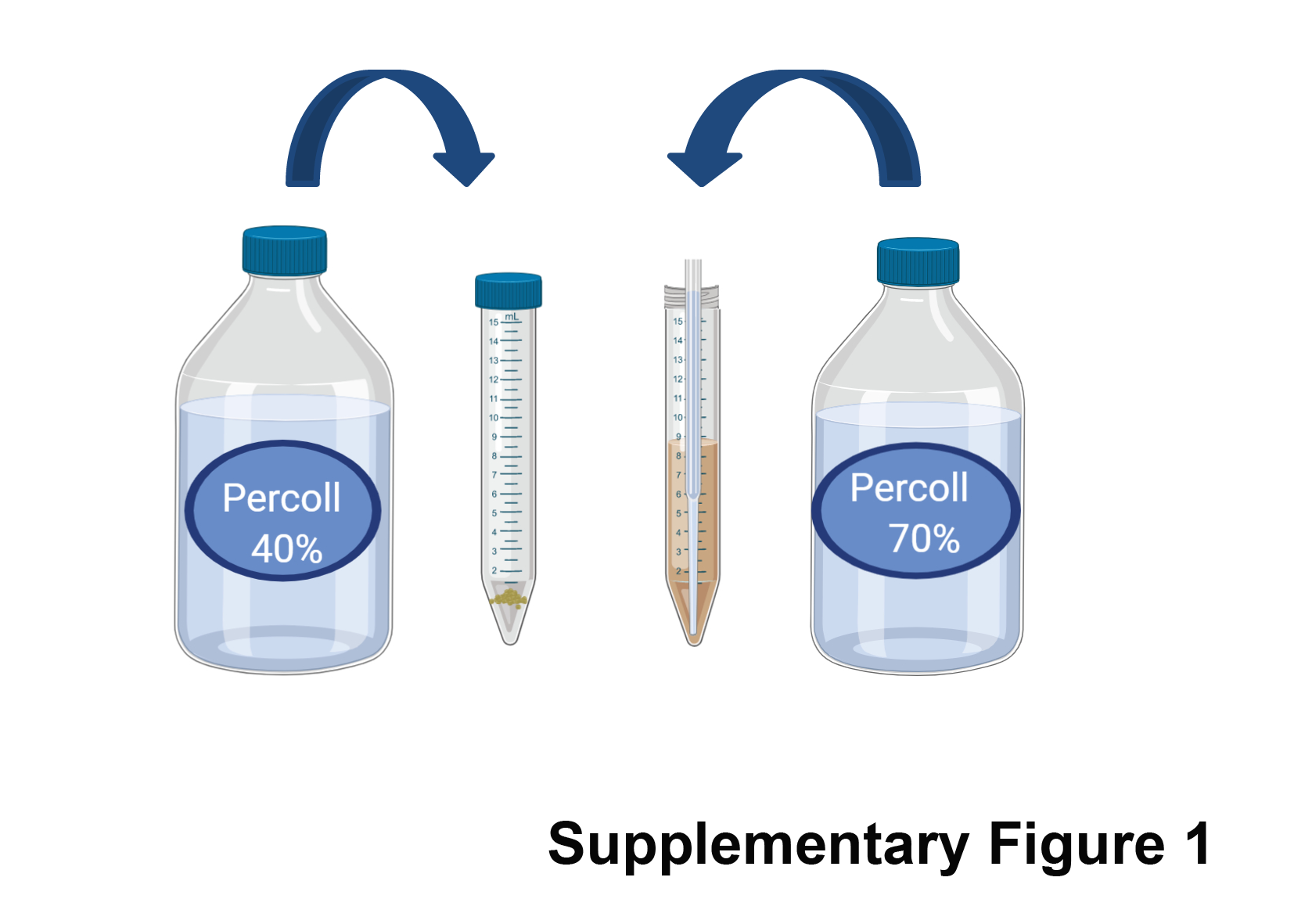

Supplement: Supplementary Figure 1 — Percoll gradient step. After the digestion step, cells can be resuspended in 4 ml of percoll 40%, and 3 ml of percoll 70% must be underlain via a glass pasteur pipette followed by a centrifugation step of 750 xg for 20 min. [file Image_1.tif]
